# The Frequent Adiponutrin (*PNPLA3*) Variant p.Ile148Met Is Associated with Early Liver Injury: Analysis of a German Pediatric Cohort

**DOI:** 10.1155/2015/205079

**Published:** 2015-08-04

**Authors:** Marcin Krawczyk, Roman Liebe, Ina B. Maier, Anna Janina Engstler, Frank Lammert, Ina Bergheim

**Affiliations:** ^1^Department of Medicine II, Saarland University Medical Center, Saarland University, 66421 Homburg, Germany; ^2^Laboratory of Metabolic Liver Diseases, Department of General, Transplant and Liver Surgery, Medical University of Warsaw, 02-091 Warsaw, Poland; ^3^Klinik für Innere Medizin II, Klinikum Mannheim, Universität Heidelberg, 68135 Mannheim, Germany; ^4^Department of Nutritional Medicine, Universität Hohenheim, 70599 Stuttgart, Germany; ^5^Department of Nutritional Sciences, SD Model Systems of Molecular Nutrition, Friedrich-Schiller-Universität, 07743 Jena, Germany

## Abstract

*Introduction.* The common adiponutrin (*PNPLA3*) variant p.Ile148Met is associated with liver injury. Here, we investigate the association of this polymorphism with hepatic and metabolic traits in a pediatric cohort. *Patients and Methods.* The study cohort comprised 142 German children (age 5–9 years, 98 overweight, 19 children with NAFLD). *Results.* Overweight children presented with increased serum ALT (*P* = 0.001) and GGT (*P* < 0.001) activities. ALT activities differed significantly (*P* = 0.02) between carriers of different *PNPLA3* genotypes in the entire study cohort, in normal weight children (*P* = 0.02) and in children younger than 7 years (*P* = 0.02). Carriers of the prosteatotic *PNPLA3* genotype p.148Met/Met displayed higher ALT activities as compared to children with the frequent genotype p.148Ile/Ile (*P* = 0.01). The BMI was however a stronger predictor of ALT activities compared to the *PNPLA3* genotype (*P* < 0.001 and *P* = 0.06, resp.). The variant was associated with increased serum glucose levels (*P* = 0.01) and HOMA index (*P* = 0.02) in carriers of the p.148Ile/Met genotype but did not affect other metabolic traits or the presence of NAFLD. *Discussion.* The frequent *PNPLA3* variant p.Ile148Met is associated with serum ALT activities already at a young age.

## 1. Introduction

The global obesity epidemic has turned nonalcoholic fatty liver disease (NAFLD) from a relatively rare phenomenon to a global health problem [1]. While steatosis, the presence of lipid droplets, is itself not necessarily harmful, it can progress into nonalcoholic steatohepatitis (NASH) in both children [2] and adult patients [3]. The distinguishing factor between simple steatosis/NAFLD and NASH is hepatocyte injury, characterized by ballooning, apoptosis, and infiltrating inflammatory cells. Between 10 and 30% of individuals with NASH may develop cirrhosis, that is, functional loss of liver tissue due to excess deposition of extracellular matrix, within 10 years. The prevalence of pediatric NALFD has been estimated as high as 10% [4], and the frequency of NAFLD in overweight and obese children may reach 25% and 76%, respectively [1]. However, as in many countries like Germany, assessment of liver status is not mandatory in children and adolescents and most available data to date were derived in clinical setting, the real figure may be even higher.

A nonsynonymous sequence variant (rs738409) of the human patatin-like phospholipase domain containing gene 3 (*PNPLA3*) resulting in the amino acid substitution p.Ile148Met has been identified as major determinant of increased liver fat content and key susceptibility factor for NAFLD in adults [5]. There have now been several studies in pediatric populations regarding the impact of rs738409 on the prevalence and progression of nonalcoholic fatty liver in childhood [6–8]. To test for an association of the* PNPLA3* rs738409 variant with liver damage in very young children, we analyzed a pediatric cohort of 142 patients aged between 5 and 9 years (described in detail in [9]) for the impact of this genetic risk factor on subclinical liver damage and NAFLD. Furthermore, we investigated the impact of this polymorphism on metabolic and anthropometric traits.

## 2. Patients and Methods

The cohort analysed in the current study comprises 142 German children (100 Caucasians, 42 Asians, 64 boys, median age 7 years, age range 5–9 years, and median standard deviation score (SDS)-body mass index (BMI) = 1.56). The participants of the study were volunteers recruited between April 2009 and December 2010 from elementary and primary schools in the greater area of Stuttgart. In addition, information leaflets asking for volunteers were distributed at children's parties and family venues and in sports clubs.

Table 1 summarizes the characteristics of the studied cohort. All pediatric patients underwent careful clinical examination including measurements of anthropometric traits. The BMI values were stratified according to reference data for German children as follows: normal weight: BMI < 90 percentile BMI and overweight: BMI > 90 percentile BMI [10]. Children with known viral and nonviral liver diseases, other than NAFLD, were excluded from the study. Moreover, children included had no known history of renal insufficiency, diabetes types 1 and 2, chronic disease of the gastrointestinal tract, or taking lipid-lowering drugs or drugs affecting lipid metabolism. NAFLD was assessed by abdominal ultrasound, which was performed in all study participants by experienced pediatricians. Presence of steatosis was graded according to the following scale: grade 0: no steatosis, grade 1: mild steatosis, grade 2: moderate steatosis, and grade 3: severe steatosis [11]. In each patient we measured anthropometric parameters, and blood samples were obtained from fasted subjects. Liver functions tests as well as serum insulin levels were determined by standard clinical-chemical assays. Levels of concurrent fasting insulin and glucose were used to estimate insulin resistance using the homeostasis model assessment (IR-HOMA) index with the following formula: insulin (mU/mL) × fasting plasma glucose (mmol/L)/22.5 [12]. Informed consent was obtained from at least one parent of each patient. The protocol of the study was approved by the Ethics Committee of the Landesärztekammer Baden-Württemberg, Stuttgart.

## 3. Genotyping and Statistics

In all individuals we genotyped the frequent* PNPLA3* variant rs738409 according to the methodology described previously [13]. Briefly, the DNeasy Blood and Tissue Kit (Qiagen) was used to isolate genomic DNA from EDTA anticoagulated blood. PCR reactions contained 20 ng DNA, 900 nM of each primer, 1 × *TaqMan* Universal Master Mix, and 200 nM of VIC-labelled and FAM-labelled probes in 25 *μ*L reactions. Following amplification conditions were set 95°C for 10 min, 40 cycles of 92°C for 15 s, and 60°C for 1 min. The quality was ensured by inclusion of negative controls and DNA samples with known* PNPLA3* genotypes as internal controls. Finally, the genotyping results were analysed with the SDS software (version 2.0.5). Consistency of genotyping results with Hardy-Weinberg equilibrium (HWE) was tested with an exact test (http://ihg.gsf.de/cgi-bin/hw/hwa1.pl). Association (case-control) analysis was performed in contingency tables (genotypes: Armitage's trend test; alleles: chi^2^ test). Kolmogorov-Smirnov's test was used to determine whether quantitative data had a normal distribution. The nonparametric analysis of variance (ANOVA) with post hoc tests was used to assess the difference in serum levels of liver enzymes and metabolic and anthropometric traits among carriers of different* PNPLA3* genotypes. Regression analyses were performed to analyse effects of the* PNPLA3* genotypes and BMI on increased serum ALT. Two-sided *P* < 0.05 was considered significant. All tests were performed with GraphPad Prism 5.0 (GraphPad Software, San Diego, USA) or SPSS 20.0 (SPSS, Munich, Germany).

## 4. Results

**Figure 1:**
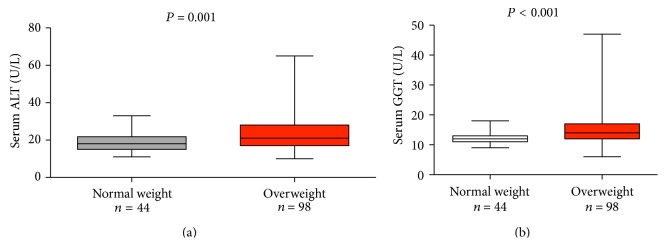
Obese children present with increased serum ALT and GGT activities. Serum ALT (a) and GGT (b) activities differed significantly (*P* = 0.001 and *P* < 0.001, resp.) between obese and normal weight children. We did not detect any significant differences between both groups with respect to the AST activities (*P* > 0.05).

**Figure 2:**
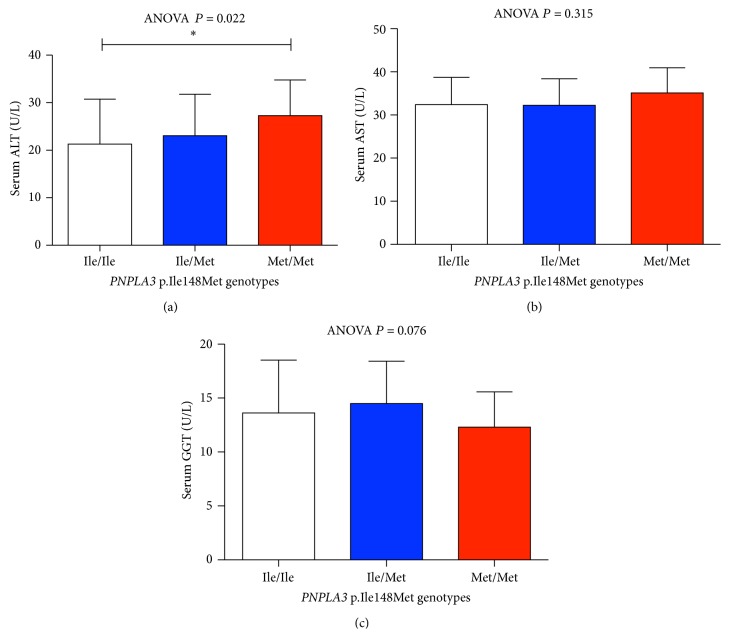
The prosteatotic* PNPLA3* variant p.Ile148Met is associated with higher serum ALT activity at young age. Serum ALT activities differ significantly (ANOVA *P* = 0.022) between carriers of different* PNPLA3* genotypes. Especially children carrying the* PNPLA3* p.148Met/Met variant present with significantly (^*∗*^ *P* = 0.009) higher ALT as compared to the carriers of the common variant* PNPLA3* p.148Ile/Ile (a). Presence of this variant was not associated with other liver function tests ((b) and (c)).

Overall, 44 children presented with normal body weight (23 boys, age range 5–9 years, median age 7 years) and 98 children were overweight (41 boys, age range 5–9 years, median age 7 years). As shown in Figures 1(a) and 1(b), obese children presented with significantly increased serum ALT and GGT activities (*P* = 0.001 and *P* < 0.001, resp.). On the other hand, we did not detect any differences in serum AST between both groups and there were no significant differences in age and gender distribution (all *P* > 0.05). In the whole cohort we observed the following genotype frequencies:* PNPLA3* p.148Ile/Ile = 75 (52.8%),* PNPLA3* p.148Ile/Met = 57 (40.1%), and* PNPLA3* p.148Met/Met = 10 (7.1%). These frequencies did not differ significantly from values presented in previous publications [14] and were in line with HWE (*P* = 0.99). Overall, there was no association between the* PNPLA3* genotype and presence of fatty liver as assessed by abdominal sonography (*N* = 19, *P* > 0.05). As shown in Figure 2(a), we detected a significant (*P* = 0.022) difference in serum ALT activities between carriers of different* PNPLA3* genotypes. In particular, homozygous risk allele carriers (i.e., with the genotype* PNPLA3* p.148Met/Met) presented with significantly (*P* = 0.009) higher median serum ALT (27 IU, range 17–40 IU) as compared to carriers of the genotype* PNPLA3* p.148Ile/Ile (median 19 IU, range 10–60 IU).

In separate analyses in normal weight and overweight children the association remained significant in the nonoverweight cohort (ANOVA *P* = 0.016): Carriers of the* PNPLA3* genotype p.148Met/Met (*N* = 5) presented with higher serum ALT activities (median 27, range 19–33) than carriers of the genotype p.148Ile/Ile (*N* = 29, median 16, range 12–32, *P* = 0.005) and to individuals with the genotype* PNPLA3* p.148Ile/Met (*N* = 10, median 17.5, range 11–28, *P* = 0.016). In univariate regression analyses, both the* PNPLA3* genotype and the BMI were associated with increased ALT (*P* = 0.049 and *P* < 0.001, resp.). In the multivariate analysis we detected a strong association between increased ALT and BMI (*P* < 0.001) and a trend for the association with variant* PNPLA3* (*P* = 0.056). To investigate whether the* PNPLA3* polymorphism has different effects on liver parameters in different age groups we stratified the cohort into two subhorts: aged 5-6 years (*n* = 41) and aged 7–9 years (*n* = 101). We detected a significant association between higher ALT activities and the* PNPLA3* risk variant in the younger (*P* = 0.017) but not in the older children. However, the latter cohort comprised significantly (*P* = 0.04) more obese individuals. We did not detect any association between the studied variant and serum activities of AST or GGT (Figures 2(b) and 2(c)). Moreover, we detected significant differences in fasting serum glucose levels and IR-HOMA between carriers of the* PNPLA3* genotypes (Table 2, ANOVA *P* = 0.01 and ANOVA *P* = 0.02, resp.), which were the highest in carriers of the p.148Met/Ile genotype. The variant was however not associated with other metabolic (Table 2) or anthropometric traits in the studied cohort (all *P* > 0.05).

## 5. Discussion

This study investigates the impact of the common* PNPLA3* variant p.Ile148Met on liver health in a cohort of German children and describes an association of the risk allele p.148Met with liver injury as indicated by higher serum ALT activities. We identify an at risk population that is likely to develop liver injury rather than a population of sick children. Indeed, only a small number of individuals in our cohort have already developed fatty liver, and increased serum ALT activities in most carriers of the risk variant stay within what is considered the normal range (Figure 2(a)). However, extrapolating from older cohorts [14–16], it is obvious that liver injury and subsequent development of clinical symptoms are likely to follow unless a change in lifestyle is pursued. Indeed, previous studies (e.g., [17]) showed that increased ALT values within the normal range are clinically relevant; moreover, it was previously postulated that the currently accepted ALT serum thresholds are set too high to detect hepatopathies in pediatric patients [18].

Prior results from studies on the impact of* PNPLA3* p.Ile148Met genotypes in pediatric cohorts were variable, probably due to differences in the groups tested. Valenti et al. [7] sampled 149 children and adolescents (mean age of 10.2 years) and observed a striking association of the* PNPLA3* p.148Met allele with NASH and fibrosis, but not with serum AST or ALT. Giudice et al. [8] tested 1058 patients aged between 2 and 16 years (mean age 10.6 years) and found an association of the* PNPLA3* prosteatotic allele with higher serum AST and ALT activities. Particularly pronounced in the subgroup with a high level of abdominal fat (waist-to-height ratio > 0.62). These data were in agreement with findings by Romeo et al. [6] in 475 obese children and adolescents (mean age 10.3 years) who showed significantly increased serum ALT and AST values for carriers of the p.148Met allele. Interestingly, a recent analysis of children aged 6–8 years showed an association between the* PNPLA3* variant and increased liver tests in obese but not in normal weight children [19]. This association even increased in the 2-year follow-up [19]. In our cohort, in contrast, the* PNPLA3* risk genotype was associated with serum ALT in normal weight children. Although overweight children carrying the p.148Met/Met variant presented with increased ALT activities, the difference in comparison to carriers of the p.148Ile/Ile genotype was not significant (*P* = 0.068). The apparent discrepancies between these observations might be related, for example, to the presence of different nongenetic triggers of liver injury (e.g., diet), which might modulate the interaction between genetic predisposition, BMI, and liver disease. Of note, our results in normal weight children are in line with a previous Mexican study analyzing a cohort of 1037 children aged 6–12 years [20]. This study [20] demonstrated that the* PNPLA3* risk variant has a stronger effect on serum ALT in normal weight children than in overweight and obese children. In our study we included children with an average age of 7 years; hence we depict a very early association between the genotype and the presence of increased serum ALT activities. On the other hand, the* PNPLA3* risk allele had a lower effect on serum transaminases as compared to increased body weight.

A meta-analysis of 16 studies confirmed the impact of the* PNPLA3* on liver fat content and the severity of NAFLD as measured by necroinflammatory scores and fibrosis stages [21]. In our cohort we did not detect any significant association between the* PNPLA3* variant and the presence of fatty liver. This might be related to the fact that only 13% of participating children had fatty liver. Our results demonstrate that hepatic injury, as reflected by increased serum transaminases, is present in young children carrying the rare* PNPLA3* variant already before fatty liver can be detected. The authors of the previous studies (reviewed in detail in [14]) hypothesized that PNPLA3 may possess both lipase and triglyceride synthase activities. The analysis [22] of the Pnpla3 p.Ile148Met knock-in mice demonstrated that the prosteatotic allele p.148Met may lead to an increased expression of PNPLA3 on lipid droplets under a high-sucrose diet. Since it is not entirely clear yet how the* PNPLA3* risk variant impacts lipase function and facilitates the cell damage evidenced by increased ALT levels, it is essential to study the resulting clinical phenotype in great detail and under various circumstances, that is, in different age groups. Interestingly, we detected significant differences in glucose levels and IR-HOMA among carriers of the* PNPLA3* genotypes (Table 2). These were highest among children with the p.148Ile/Met genotype, which confers an intermediate NAFLD-risk. The impact of the* PNPLA3* genotype on insulin resistance is controversial. Although in most published studies no association between the* PNPLA3* variant and insulin resistance or glucose levels was detected [14], a few studies reported significant effects of the* PNPLA3* variant on metabolic status [23–26]. The weaknesses of our study include a relatively small sample size and a lack of liver biopsy or MRI as measurements of hepatic steatosis. MRI would also allow analysis of body fat composition and investigate its interactions between the* PNPLA3* genotype, body fat distribution, and liver injury.

## 6. Conclusion

Our data from a relatively small German pediatric cohort suggest that young children bearing the homozygous risk allele of* PNPLA3* p.Ile148Met suffer from liver damage at an early age. We did not detect any association between the studied variant and the presence of other traits that are commonly linked to increased hepatic fat accumulation, apart from increased glucose levels and IR-HOMA in individuals with the Ile/Met variant. Hence, at risk pediatric patients may be easily missed when increased BMI is used as a cut-off mark for screening individuals at risk of developing NAFLD. Thus, screening of children for the* PNPLA3* risk allele regardless of their body weight may be useful in regards to an early determination of “at-risk” children. While we are aware of the risk of stigmatising children by subjecting them to genotyping and allocating them to an “at-risk” group, it is our view that the benefit derived from informing parents in a confidential and thorough manner will outweigh any such risk. Further studies are urgently needed to develop tailored genotype-guided strategies for the prevention of NAFLD and its consequences in children.

## Figures and Tables

**Table 1:** Clinical characteristics of the pediatric cohort.

Variables	Subject characteristics
*N* (male/female)	142 (64/78)
Age (years)	7 (5–9)^&^
Patients with NAFLD^*^ (*N*)	19
Overweight individuals (*N*)	98
BMI-SDS	1.56 (−1.09–3.46)^&^

^&^Values are given as medians and ranges. ^*^Detected by abdominal ultrasonography.

BMI-SDS: body mass index-standard deviation score; NAFLD: nonalcoholic fatty liver disease.

**Table 2:** Metabolic traits in relation to the *PNPLA3* p.Ile148Met genotypes.

Feature	*PNPLA3* p.Ile148Met genotype			*P* value
	Ile/Ile (*n* = 75)	Ile/Met (*n* = 57)	Met/Met (*n* = 10)	
Triglycerides (mg/dL)	70.0 (25.0–294.0)	69.0 (35.0–231.0)	57.5 (41.0–92.0)	0.39
HDL cholesterol (mg/dL)	54.0 (26.0–71.0)	52.0 (21.0–77.0)	50.5 (38.0–64.0)	0.55
LDL cholesterol (mg/dL)	108.0 (63.0–155.0)	114.0 (62.0–166.0)	99.5 (93.0–124.0)	0.09
Total cholesterol (mg/dL)	176.0 (113.0–230.0)	185.0 (121.0–245.0)	164.5 (145.0–187.0)	0.06
Glucose (mg/dL)	84.5 (72.0–103.0)	87.0 (63.0–122.0)	84.5 (75.0–94.0)	0.01
Insulin (*µ*lU/mL)	10.0 (4.1–38.8)	11.0 (1.9–33.7)	8.4 (2.8–17.0)	0.06
IR-HOMA	2.0 (1.0–9.2)	2.6 (0.5–7.7)	1.6 (0.6–3.6)	0.02

Ile: isoleucine; IR-HOMA: insulin resistance homeostasis model assessment; Met: methionine; p: protein (amino acid number); *PNPLA3*: adiponutrin. Values are given as medians and ranges.

## List of Abbreviations

ANOVA: Analysis of variance
BMI-SDS: Body mass index-standard deviation score
HWE: Hardy-Weinberg equilibrium
IR-HOMA: Insulin resistance homeostasis model assessment
NAFLD: Nonalcoholic fatty liver disease
NASH: Nonalcoholic steatohepatitis
PNPLA3: Patatin-like phospholipase domain containing 3 (adiponutrin).
